# Prevalence of Overweight, Obesity, and Abdominal Obesity among Urban Saudi Adolescents: Gender and Regional Variations

**Published:** 2014-12

**Authors:** Hazzaa M. Al-Hazzaa, Nada A. Abahussain, Hana I. Al-Sobayel, Dina M. Qahwaji, Nouf A. Alsulaiman, Abdulrahman O. Musaiger

**Affiliations:** ^1^Pediatric Exercise Physiology Research Laboratory, College of Education, King Saud University, Riyadh, Saudi Arabia; ^2^Obesity Research Chair, King Saud University, Riyadh, Saudi Arabia; ^3^School Health, Ministry of Education, Eastern Province, Saudi Arabia; ^4^Department of Rehabilitation Sciences, College of Applied Medical Sciences, King Saud University, Riyadh, Saudi Arabia; ^5^Department of Clinical Nutrition, College of Applied Medical Sciences, King Abdulaziz University, Jeddah, Saudi Arabia; ^6^PO Box 8342, Jeddah, 21482, Saudi Arabia; ^7^Arab Center for Nutrition, Manama, Bahrain, and Nutrition and Health Studies Unit, Scientific Research, University of Bahrain, Bahrain

**Keywords:** Adolescents, BMI, Obesity, Waist-circumference, Saudi Arabia

## Abstract

The nutrition transition with associated lifestyle-related non-communicable diseases has rapidly reached many developing countries, including Saudi Arabia. Therefore, the objective of this study was to examine the prevalence of overweight, obesity, and abdominal obesity among Saudi adolescents. This school-based multicentre cross-sectional study was conducted during 2009-2010 in three major cities in Saudi Arabia: Al-Khobar, Jeddah, and Riyadh. Participants included 2,908 students of secondary schools (1,401 males and 1,507 females) aged 14 to 19 years, randomly selected using a multistage stratified cluster-sampling technique. Weight, height, and waist-circumference were measured; prevalence of overweight and obesity was determined using age- and sex-specific BMI cutoff reference standards of the International Obesity Task Force (IOTF). Abdominal obesity was determined using waist-to-height ratio (WHtR) cutoffs (above 0.5). The prevalence of overweight was 19.5% in males and 20.8% in females while that of obesity was 24.1% in males and 14% in females. The prevalence of abdominal obesity in males and females was 35.9% and 30.3% respectively. Higher prevalence of obesity was observed among adolescents in private schools. Across all ages, overweight and obesity ranged from 39.9% to 45.6% in males and from 30.4% to 38.7% in females. ANCOVA, controlling for age, showed significant interaction effects (city by gender). It is concluded that the proportions of overweight, obesity, and abdominal obesity, observed among Saudi adolescents were remarkably high. Such high prevalence of overweight and obesity is a major public-health concern.

## INTRODUCTION

In recent years, paediatric obesity has increased considerably across the developed and developing countries (1). This has prompted World Health Organization (WHO) to designate obesity as one of the most important public-health threats (2,3). Indeed, childhood obesity is well-recognized to associate with co-morbidities (4-6). Metabolic complications associated with obesity during childhood increase the risk of type 2 diabetes and early cardiovascular disease (7). Furthermore, obesity in adolescence was shown to correlate significantly with increased risk of severe obesity in adulthood (8). Obesity may also affect psychological health as obese children are more likely to report low self-esteem compared to their non-obese peers (9).

Nutrition transition with associated lifestyle-related non-communicable diseases, which was first observed in the developed countries, has rapidly reached many developing countries, including Saudi Arabia (10-12). In fact, during the past three decades, the kingdom of Saudi Arabia has undergone enormous lifestyle-related transformation, which has largely contributed to the increase in the prevalence of obesity observed among Saudi children and youths (10). Recent national estimates of the prevalence of obesity (based on body mass index from data collected in 2005) indicated that overweight and obesity rates among Saudi adolescents aged 13-18 years were 26.6% and 10.6% respectively (13). Furthermore, while the prevalence of high BMI among children and adolescents in some of the developed countries, like the United States, is showing a plateau between 1999 and 2008 (14), the prevalence of obesity among Saudi children and adolescents is still increasing. Evidence from serial cross-sectional assessments carried out over the years, on Saudi children and adolescents, using BMI (or fat percentage), showed a noticeable rise in obesity level over the past two decades (15-17). Because of this rapid increase in childhood obesity in Saudi Arabia over the past years, data from even a recent prevalence study a few years back may be considered outdated.

Abdominal obesity, which represents both subcutaneous and visceral fat accumulation, has been linked to increased cardiometabolic risks in children and adolescents (18,19). The use of waist-circumference (WC) and waist-to-height ratio (WHtR) for determining abdominal obesity in children was shown to be simple, sensitive, and specific (20,21). WC and WHtR also appeared to be better predictors of cardiometabolic disease risk in children than is BMI (20,22-24). Despite the usefulness of WC and WHtR in assessing central obesity, only one previous local study (25) employed WC while none has used WHtR in studying obesity among Saudi children and adolescents. Therefore, the objective of this cross-sectional comparative study was to provide recent estimates of the prevalence of overweight and obesity, using BMI cutoff standards as well as the levels of abdominal obesity, using WC and WHtR, in representative samples of Saudi adolescents from three major cities in Saudi Arabia, namely Riyadh, Al-Khobar, and Jeddah.

## MATERIALS AND METHODS

### Selection of participants

The present study is a part of the Arab Teens Lifestyle Study (ATLS). The ATLS is a school-based cross-sectional multicentre collaborative study (26). The sampling technique and the methodology were fully described in previous publications (26-28). Briefly, the sample was drawn from adolescent males and females enrolled in the secondary schools in three major cities of Saudi Arabia; Riyadh, Jeddah, and Al-Khobar, that are located in the central, western and eastern regions of Saudi Arabia respectively. The three cities represent three different regions in Saudi Arabia and are all considered cosmopolitan cites. The minimum needed sample-size in each city was determined so that the sample proportion would be within ±0.05 of the population proportion with a 95% confidence level. The population proportion has been assumed to be 0.50 as this magnitude yields the maximum possible sample-size required. The random selection of the sample was based on a multistage stratified cluster-sampling technique. At the first stage, a systematic random-sampling procedure was used in order to select the schools. The schools were stratified into secondary schools for boys and girls, with further stratification into public and private schools for boys and girls. The selection of the private/public schools was proportional to size. Four schools (two each from boys and girls schools) were selected from each of the four geographical areas of each city (East, North, South, and West). At the second stage, classes were chosen from each grade (level), using simple random-sampling design. In this way, one class was randomly selected in each grade of the three grades (Grade 10, 11, and 12) in each secondary school. Thus, we had a total selection of at least 24 classes in each city (12 each from boys and girls schools). All students in the selected classes, who were free from any physical abnormality, were invited to participate in the study. Due to the differences in class-size from city to city and from private to public schools, the sample-sizes for the participating cities were varied. The data collection occurred during 2009-2010. The study protocol and procedures were approved by the Board of Educational Research Center at King Saud University as well as by the General Directorate of Education in each of the respective cities. In addition, we obtained approval from school authorities and parental consents for conducting the survey. The total sample-size consisted of 2,908 adolescents, comprising 1,401 males and 1,507 females aged between 14 and 19 years.

### Anthropometric measurements

A trained researcher performed the anthropometric measurements in the morning and according to standardized procedures. Weight was measured to the nearest 100 g, using calibrated portable scale. Measurements were done with minimal clothing and without shoes. Height was measured to the nearest cm, without shoes, using a calibrated measuring rod. Body mass index (BMI) was calculated as a ratio of weight in kg by height in metre squared. The age- and sex-specific BMI cutoff reference standards of the International Obesity Task Force (IOTF) were used in identifying overweight and obesity in adolescents between the age of 14 and 17 years (29). For participants aged 18 years and above, we used cutoff points of 25-29.9 kg/m^2^ for overweight and 30 kg/m^2^ and higher for obesity. Waist-circumference (WC) was measured horizontally to the nearest 0.1 cm at the level of umbilicus, using a non-stretchable measuring tape. Participants were measured in private at an examination site in each school. For cultural reason, WC was measured in girls with light shirt on, and this was later adjusted (corrected) using a correction factor of −1 cm, based on the results of a small pilot study that we have previously conducted. Waist-to-height ratio (WHtR) was calculated, dividing WC (in cm) by height (in cm). A WHtR cutoff point of 0.50 was used in defining abdominal obesity in males and females (20,22).

### Statistical analysis

Data were checked and entered into a computer, using standardized entry codes written on an SPSS data file. Data analysis was performed using SPSS (version 15), and descriptive statistics are presented as mean±standard deviation or proportions. Chi-square tests were used in testing the associations between the proportions of overweight and obesity, based on BMI cutoffs as well as abdominal obesity, based on WHtR, across genders and regions. Differences in anthropometric measurements between the age of 15 and 19 years were tested using one-way ANOVA, with Bonferroni test. Cross-tabulations, with chi-square tests, of BMI categories and WHtR categories were also performed. In addition, we used a two-way ANCOVA (2 by 3) while controlling for the effects of age to test the differences in each of BMI, WC, and WHtR across genders (male and female) and cities (Al-Khobar, Jeddah, and Riyadh). The level of significance was set at a p value of less than 0.05.

## RESULTS

The descriptive characteristics of the participants, grouped by gender and city are shown in Table 1. The number of females in the sample slightly exceeded that of the male participants (51.8% vs 48.2%). Mean values for age of the males and females were 16.7 and 16.5 years respectively. For the whole sample, males showed significantly higher values for age, weight, height, BMI, and WC but not for WHtR. There were significant (p<0.05) differences between females from the three cities in height, BMI, WC, and WHtR while WC, relative to city, was significantly (p<0.05) different among both males and females. Adolescent males in Riyadh significantly (p<0.05) exhibited the lowest WC and WHtR compared to Al-Khobar and Jeddah whereas adolescent females in Al-Khobar showed the lowest WC and WHtR compared to those in Riyadh and Jeddah (p<0.05). The Figure presents results of the two-way ANCOVA while controlling for the effect of age. Values for BMI, WC, and WHtR clearly exhibited significant interaction effects (city by gender) as p values ranged from 0.012 to <0.001.

**Table 1.** Descriptive characteristics of the participants. Data are mean±standard deviation (N=2,908 adolescents)

Variable	Al-Khobar		Jeddah		Riyadh		All	
	Male	Female	Male	Female	Male	Female	Male	Female
Number of subjects	348	367	560	632	493	508	1,401	1,507
Age (years)^a,c^	16.8±1.1	16.6±1.0	16.8±1.0	16.5±1.1	16.6±1.2	16.5±1.0	16.7±1.1	16.5±1.1
Weight (kg)^b,c^	69.9±20.8	57.2±13.9	71.4±20.7	57.4±15.7	68.3±19.9	59.2±16.1	70.0±20.5	57.9±15.5
Height (cm)^b,c^	168.7±7.9	157.7±6.1	168.4±6.6	155.9±5.8	168.1±7.3	156.8±5.8	168.4±7.2	156.7±5.9
BMI (kg/m^2^)^b,c^	24.5±6.8	23.0±5.4	25.1±6.8	23.6±6.3	24.1±6.6	24.0±6.2	24.6±6.7	23.6±6.1
Waist-circumference (cm)^a,b,c^	80.9±17.0	71.3±11.9	80.8±15.0	75.8±13.0	77.6±14.6	74.2±13.7	79.7±15.4	74.2±13.1
Waist-height ratio (WHtR)^a,b^	0.479±0.09	0.452±0.97	0.480±0.09	0.486±0.08	0.462±0.08	0.474±0.09	0.474±0.09	0.474±0.08

One-way ANOVA; ^a^ and

^b^Significant difference between cities at p<0.05 for males and females respectively; *t*-tests for independent samples:

^c^Significant gender difference for the whole sample at p<0.05

Based on the IOTF cutoff points for BMI, the prevalence of overweight was 19.5% and 20.8% for males and females respectively while the prevalence of obesity was 24.1% and 14% for males and females respectively (Table 2). When using cutoffs based on WHtR of 50%, the prevalence of abdominal obesity in males and females was 35.9% and 30.3% respectively. The prevalence rate of combined overweight and obesity, as shown in Table 2, revealed that males in Jeddah ranked the highest among all adolescent males while females in Riyadh were ranked slightly higher than females in Jeddah and Al-Khobar. As to the proportion of adolescents above 50% of WHtR, males in Riyadh and females in Al-Khobar showed the lowest percentages among all males and females respectively. In addition, further analyses showed that adolescents from private schools had higher prevalence of obesity (22.7% vs 17.4%, p<0.001) and abdominal obesity (38.1% vs 31.1%, p<0.001).

Table 3 shows the results of anthropometric measurements and the prevalence of combined overweight and obesity relative to age from 15 to 19 years. Data for age 14 years were not included in Table 3 because there were much fewer cases in this age-group compared to the other age-groups. Analysis revealed that only there were significant differences in height and WC between age-groups. In addition, combined overweight and obesity ranged from 39.9% to 45.6% in males and from 30.4% to 38.7% in females. The prevalence of overweight and obesity also showed a downward trend among males with advancing ages, except at age 19 years where there was an increase in the overweight and obesity rate while there was an upward trend among females with increasing ages from 15 to 19 years.

Table 4 presents the cross-tabulations of BMI-based classification (normal, overweight, and obese) against WHtR categories (below and above 50%). Chi-square tests as well as contingency coefficients indicated significant associations between these proportions in males and females across obesity indices (p<0.001), indicating consistent findings for the measures of obesity and abdominal obesity among males and females in the present study. Finally, Table 5 provides comparisons of overweight and obesity prevalence data from the current study with those prevalence rates that were previously published on Saudi adolescents (13,25,30-39).

## DISCUSSION

The present study provided a unique comparative analysis of the prevalence of obesity among Saudi adolescents in three major cities of Saudi Arabia, a country undergoing rapid lifestyle transition. The findings from the current study confirmed high prevalence of overweight and obesity among Saudi adolescents observed in previous studies and reported WHtR prevalence data for the first time. Obesity has now become a global epidemic and is no longer limited only to the developed countries of the world (1,2).

Cross-sectional comparisons of the prevalence of overweight and obesity in youths from different studies are somewhat challenging. This is due to many reasons, including the use of non-representative samples, differences in age and gender of the samples, and the use of different BMI cutoff reference standards. As presented in Table 5, the reported studies indicated wide variations in the prevalence of overweight and obesity among Saudi adolescents. However, except for the samples from Abha, which is a city with high altitude, most recent studies indicated high prevalence of overweight and obesity. The prevalence of overweight and obesity reported in the present study appeared slightly different from what was reported in a national study conducted in 2005 and published recently (13). There are several reasons for these differences in the prevalence of obesity. First, the present study is more recent than the national study. Second, our sample came from major cities in Saudi Arabia where most changes in lifestyle are occurring while sample of the national study came from both urban and rural areas. Third, the present study used IOTF reference standards for determining the prevalence of overweight and obesity while the national study used WHO reference standards for the same purpose. Finally, our study included adolescents from 14 to 19 years of age while the national study presented the prevalence data for 13-18 years age-group.

**Figure.**
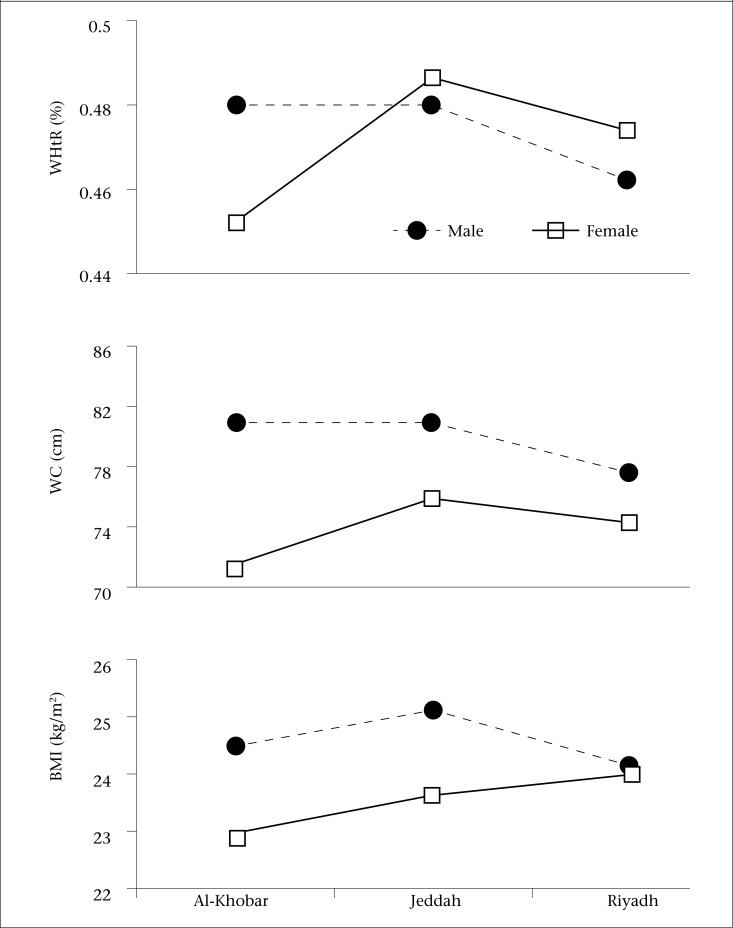
Body mass Index (BMI) in kg/m^2^, waist-circumference (WC) in cm and waist-to-height ratio (WHtR) in percentage points for the Saudi adolescents relative to city and gender. Results of two-way ANCOVA, controlling for the effect of age, indicate that, for BMI, a significant interaction (city by gender; p=0.012) and significant main effects for gender (p<0.001) but not for the city (p=0.115); for WC: a significant interaction (city by gender; p<0.001) and significant main effects for city (p< 0.001) and for gender (p< 0.001); for WHtR: a significant interaction (city by gender; p<0.001) and significant main effects for city (p<0.001) but not for gender (p=0.395)

**Table 2.** Proportions (%) of Saudi adolescents within specific cutoff values for body mass index (BMI) and waist-to-height ratio (WHtR)

Category	Al-Khobar		Jeddah		Riyadh		All	
	Male	Female	Male	Female	Male	Female	Male	Female
Cutoff values based on BMI^a^								
Overweight	20.7	21.5	19.6	19.3	18.5	22.0	19.5	20.8
Obese	22.1	11.6	26.5	15.5	22.7	13.8	24.1	14.0
Overweight or obese	42.8	33.1	46.1	34.8	41.2	35.8	43.6	34.8
Cutoff values based on WHtR^b^								
At or above 50% of WHtR	38.4	23.9	37.6	38.0	32.3	28.2	35.9	30.3
Below 50% of WHtR	61.6	76.1	62.4	62.0	67.7	74.7	64.1	69.7

^a^Chi-square tests across cities: p=0.409 for males and 0.410 for females;

^b^Chi-square tests across cities: p=0.106 for males; p<0.001 for females

**Table 3.** Anthropometric measures and prevalence of overweight plus obesity in Saudi male and female adolescents relative to age (data are mean±standard deviation and percentages)

Variable	Gender	Age in years				
		15	16	17	18	19
Number of participants	M	180	404	451	288	61
	F	219	533	484	204	40
Weight (kg)	M	66.4±20.0	68.8±19.6	71.2±20.4	72.1±22.8	70.3±19.5
	F	56.6±14.3	58.7±16.5	57.1±14.0	59.4±16.4	61.2±20.7
Height (cm)	M^a^	166.4±7.8	167.2±7.1	169.6±6.9	169.5±6.9	167.6±6.0
	F	157.1± 5.7	156.4±5.8	156.9±5.8	156.4±6.2	156.2±7.4
BMI (kg/m^2^)	M	23.8±5.9	24.5±6.4	24.7±6.7	25.1±7.6	25.0±6.8
	F	22.9±5.6	23.9±6.3	23.2±5.6	24.2±6.6	25.0±7.9
WC (cm)	M^b^	77.1±14.9	79.4±15.0	80.1±15.3	81.8±16.6	78.7±14.9
	F	73.9±12.1	74.6±13.5	73.0±12.9	75.6±12.9	77.7±16.4
WHtR	M	0.46±0.08	0.48±0.09	0.47±0.09	0.48±0.10	0.47.0±0.09
	F	0.47±0.0.08	0.48±0.09	0.47±0.08	0.48±0.08	0.50±0.011
Overweight + obesity (%)	M^c^	45.6	45.4	43.1	39.9	46.0
	F^c^	34.3	36.8	30.4	38.7	42.5

^a^Bonferroni test results: height at age 15 years are significantly different from those at age 17 years (p<0.001) and at age 18 years (p<0.001); Heights at age 16 years are significantly different from those at age 17 years (p<0.000) and at age 18 years (p<0.001);

^b^Bonferroni test results: WC value at age 15 years is significantly (p<0.028) different from that at age 18 years;

^c^Chi-square test results p=0.579 for males; p=0.287 for females

In the present study, males had higher prevalence of combined overweight and obesity compared to females (43.6% vs 34.8%). Previous local studies, as seen in Table 5, showed inconsistent results regarding the gender difference and obesity prevalence among Saudi adolescents. Papers published earlier implied that females appeared to be more overweight and obese than males while more recent studies indicated almost the opposite trend. A recent review paper suggests that the gender differences in obesity rates are generally small and inconsistent (40). However, besides differences in lifestyles between boys and girls, it is likely that adolescent girls at this age might be more concerned with their physical appearance and would probably desire a slim body than boys.

The current study revealed some regional differences in overweight, obesity, and abdominal obesity (as judged by the WHtR data). Such differences may be attributed to factors, such as differing ethnic backgrounds and socioeconomic status (SES) of the adolescents. Although SES was not assessed in the present study, a Canadian study conducted on 7-13 years old children found that the risk of being overweight was more related to geography (province) than demographic variables, such as family income and background (41). Differences in lifestyle factors may have also influenced differences in the regional obesity rates. Indeed, total physical activity in METs-min per week as well as several eating habits, like intake of breakfast, milk, and sugar-sweetened drinks, were shown to be significantly different among adolescents living in the three regions (27).

**Table 4.** Cross-tabulation of body mass index (BMI) and waist-to-height ratio (WHtR) for Saudi adolescents (values are %)

WHtR	BMI		
	Normal	Overweight	Obese
Male^a^ (n=1,394)			
Below 50% of WHtR	83.8	14.5	1.7
At or above 50% of WHtR	7.4	28.1	64.5
Female^b^ (n=1,498)			
Below 50% of WHtR	85.7	12.6	1.6
At or above 50% of WHtR	18.5	39.2	42.3

^a^Chi-square tests and contingency coefficients for males are all significant at p=0.001;

^b^Chi-square tests and contingency coefficients for females are all significant at p=0.001

The findings of the present study showed a higher prevalence of overweight and obesity in adolescents attending private schools compared to those attending public schools. This finding could be due to the fact that adolescents in private schools came usually from families with higher socioeconomic status (higher income and educational level). At private schools, they might also have less restriction on food and snack choices compared to those in public schools. It is worth mentioning that studies on socioeconomic status and obesity suggest higher rate of obesity among low-income groups in developed countries and high-income groups in developing countries (1). Such finding agrees with the results of the present study.

The high prevalence of combined overweight and obesity (38.9%) reported in this study for the Saudi adolescents is considered even higher than the recent prevalence rate that was reported for adolescents aged 12-19 years in the United States. The National Health and Nutrition Examination Survey (NHANES) 2007-2008 reported a 34.2% prevalence of overweight and obesity (BMI equal or above sex- and age-specific 85th percentile from the 2000 CDC growth charts) among US adolescents (14). A recent international comparative study on the prevalence of overweight and obesity in school-age youths from 34 countries used IOTF reference standards and reported high prevalence of overweight and obesity for youths from countries located in North America (from 19.3% in Canada to 25.1% in the USA), Great Britain (from 18.4 in England to 21.5% in Wales), and South-Western Europe (from 18% in Portugal to 18.8% in Spain) (42). The prevalence rates of overweight and obesity for the previous study, however, were still lower than what was reported for Saudi adolescents in the present study.

The present study found considerably high prevalence rates of abdominal obesity based on cutoff above 50% of WHtR (35.9% and 30.3% for males and females respectively). The use of WHtR has the advantage of not requiring population-specific reference values or age and sex cutoff points; so, it can perfectly track changes across ages in children and adolescents (22). WHtR correlates highly with visceral fat mass (19). Such high rates of central obesity among Saudi adolescents should represent greater concern because of its association with risk factors of cardiovascular disease in children (23). In addition, greater trunk-fat values, coupled with high BMI in boys, were shown to increase the risk of central obesity in adulthood (43). Local data on WC of adolescents are scarce (25). Comparison of our mean WC results with those values recently reported on Saudi adolescents from Riyadh indicates that the males in the present study appeared to have slightly lower values while the females in our study had somewhat higher values compared to those reported for adolescents of similar age-groups (25). Comparison of our WC values with the existing international data indicates that the adolescents in the present study had higher mean values than those reported for Australian adolescents (44) and higher 50th and 95th percentiles than what have been reported on Turkish adolescents aged 15-18 years (45). However, mean values for WC and WHtR in the present study showed lower than those reported on adolescents aged 18-19 years in the United States (46).

**Table 5.** Comparison of obesity prevalence in the present study with those from previous local studies

Reference (Ref. No.)	City or region	Sample- size	Obesity definition	Age (years)	Overweight (%)		Obesity (%)		Overweight+obesity	
					Male	Female	Male	Female	Male	Female
Present study	3 major cities	2,908	IOTF	14-19	19.5	20.8	24.1	14	43.6	34.8
El Mouzan *et al.,* 2010 (13)	National	7,251	WHO	13-18	24.8	28.4	11.2	10	36	38.4
Al-Doussary *et al.,* 2010 (38)	Eastern Province	1,609	CDC	14-18	18	20.3	26.4	19.3	44.4	39.6
Collison *et al.,* 2010 (25)	Riyadh	6,078	CDC	14-16 17-19	14.3 13.0	17.2 17.7	28.2 29.7	16.8 13.3	42.5 42.7	34 31
Mahfouz *et al.,* 2008 (31)	Abha	2,696	WHO	11-19	11	-	5	-	16	-
Al-Saeed *et al.,* 2007 (32)	Al-Khobar	151	IOTF	15-17	-	27.8	-	13.9	-	41.7
Farghaly *et al*., 2007 (33)	Abha	98	WHO	16.5	8.7	13.8	11.6	20.7	20.3	34.5
Al-Almaie, 2005 (34)	Al-Khobar	1,766	IOTF	14-19	14.1	20.2	16.7	10.9	30.8	31.1
Al-Rukban, 2003 (36)	Riyadh	894	WHO	12-20	13.8	-	20.5	-	34.3	-
Abalkhail *et al.,* 2002 (37)	Jeddah	1,801	WHO	13-15 16-21	13.7 14.9		15 13.7		28.7 28.6	
El-Hazmi and Warsi, 2002 (37)	National	5,842	IOTF	12-18	14.5	15.6	5.8	6.9	20.3	22.5
Abahussain *et al.,* 1999 (38)	Al-Khobar	676	WHO	12-19	-	-	-	-	-	28.1
Al-Nuaim *et al*., 1996 (39)	National	3,223	NCHS/CDC	14-18	11.3	-	20.5	-	31.8	-

CDC=Centers for Disease Control and Prevention Reference Standards, 2000; IOTF=International Obesity Task Force Reference Standards, 2000; NCHS/CDC=National Center for Health Statistics/CDC 1977; WHO=World Health Organization Reference Standards, 1995 and 2007

Obesity is a complex disease with genetic and lifestyle factors, both playing important roles in determining a child's weight and body composition. However, despite the strong influence of genetic determinants on obesity, the genetic composition of the population does not change rapidly. Thus, the high prevalence rates of overweight and obesity seen in Saudi adolescents must reflect major changes in lifestyle-related factors, namely lack of physical activity, increased sedentary behaviours, unhealthy eating habits, or a combination of any of these factors (10,11,27,28,47). Indeed, previous research has shown that lifestyle-related behaviours that are associated with obesity among school children included increased TV-viewing, use of computers and the Internet, decreased physical activity inside and outside schools, and consumption of breakfast and sugary drinks (28,48,49). In addition, sleeping duration in adolescents has been linked to increased risk of being obese (50,51).

Lifestyle-related factors, including the changes in food patterns of the people living in this region, may have played important roles in creating obesogenic environment for Saudi children and adolescents (47). Hot climate and high air pollution in major cities of Saudi Arabia discourage outdoor activities and may increase prevalence of inactivity. Recent data indicate that 60% of children and 71% of Saudi youths do not engage in physical activity of sufficient duration and frequency (10,52). Major factors that contributed to youths’ inactivity in Saudi Arabia include a reliance on cars rather than walking for short-distance travel, including trips to and from schools (53). Insufficient vigorous physical activity was shown to be a risk factor of higher BMI for adolescent boys and girls (54). Findings from a cross-sectional survey involving youths aged 10-16 years from 34 countries demonstrated that physical activity levels were lower and television-viewing times were higher in overweight youths compared to youths with normal weights (42).

The samples in the present study came from three modern urbanized areas in Saudi Arabia. Urbanization has been suggested as an important risk factor of obesity in developing countries undergoing economic transition (55). Prior to the recent economic growth surge in the late seventies of the twentieth century, communities in major cities in Saudi Arabia were designed to support pedestrian travel in common daily activities, such as shopping, travelling to schools and mosques. Such traditional neighbourhoods are characterized by high-density houses, close-by stores, and narrowed streets that provide direct routes from place to place. These traditional design settings facilitate walking and cycling resulting in an increase in active daily living. Walking and cycling, to and from schools were common then, and this is of particular importance because both require substantial energy expenditure on regular basis. In contrast, Saudi Arabian cities are now modernized with large street networks and separate zoning for residential and commercial areas. This kind of design requires the use of automobile for every trip and totally discourages walking.

### Strengths and limitations

The findings of the present study should be seen in light of their strengths and limitations. This is a multicentre school-based study that used a representative and large sample. The study had a very high participation rate. We also used two indicators of obesity, namely cutoffs for BMI and for WHtR. One of the limitations of the study, however, was the lack of pubertal indicators, so to adjust the prevalence of overweight and obesity to the adolescent's pubertal stage. Overweight girls were shown to be associated with earlier maturation while early-mature boys were correlated with reduced BMI (56). Thus, possible differences in sexual maturational patterns between the study participants, relative to age and city, cannot be completely ruled out. However, majority of the adolescents in our sample were 15 years of age or older, something that might reduce the variations in pubertal stages to a greater extent.

### Conclusions

The high prevalence of overweight and obesity among Saudi adolescents seen in the present study is of major public-health concern and should make a strong case for greater efforts to be directed at prevention and treatment of childhood obesity in Saudi Arabia. If no drastic measures were taken to reduce the level of obesity among Saudi children and youths, we may likely experience a fair reduction in the absolute life-expectancy for the young generation. To combat childhood obesity epidemic in this part of the world, fundamental changes in public policies, the food and built environments, and health systems are required. Primary prevention through promotion of a healthful diet and active lifestyle should be the national priority in public-health policy.

**Conflict of interests:** The authors declare that they have no competing interests.

## ACKNOWLEDGEMENTS

Professor Hazzaa M AL-Hazzaa's research was supported by fund from the Educational Research Center, Deanship of Research, King Saud University. The authors also acknowledge the assistance of many male and female research assistants who assisted in the data collection throughout the participating cities.
